# Supplementary material for: Evaluation of Quantitative Platforms for Single Target Mass Spectrometry Imaging
Source: Pharmaceuticals (Basel). 2022 Sep 23;15(10):1180. doi: 10.3390/ph15101180 (PMC9609477; doi:10.3390/ph15101180)
Supplement: Supplementary file 1 [file pharmaceuticals-15-01180-s001.zip › pharmaceuticals-1909070-supplementary.pdf]

Supplementary Table S1. Limits of Blank and Limits of Detection. Complete tests of 5 imaging platforms for limit of blank (LoB) and limit of detection (LoD). Three different matrices were tested, and 3 potential cations were considered: protonated, sodiated, and potassiated. Cations that do not appear on a specific platform are omitted. Absorption mode experiments on the FT-ICR system were added later and only tested with DHB. AP-MALDI 6500+ experiments were added later and only tested with FleX matrix.

Target	Platform	Matrix	Cation	R^2	LoB (µg/g)	LoD (µg/g)
**Felodipine**	AP-MALDI 6500	DHA	H	0.9721	160.75	390.61
**Felodipine**	AP-MALDI 6500	DHB	H	0.9757	43.57	111.83
**Felodipine**	AP-MALDI 6500	FleX	H	0.5984	686.19	1150.33
**Felodipine**	AP-MALDI 6500+	FleX	H	0.9960	20.37	53.50
**Felodipine**	DESI Orbitrap	N/A	Na	0.9948	0.00	1968.17
**Felodipine**	DESI Orbitrap	N/A	K	0.9918	0.00	2629.05
**Felodipine**	FT-ICR Absorption	DHB	H	0.963	1516.33	3108.88
**Felodipine**	FT-ICR Absorption	DHB	K	0.972	5288.87	10590.57
**Felodipine**	FT-ICR Absorption	DHB	Na	0.928	5193.83	11101.11
**Felodipine**	FT-ICR Absorption CASI	DHB	H	0.982	470.38	982.17
**Felodipine**	FT-ICR Absorption CASI	DHB	K	0.984	696.11	1449.50
**Felodipine**	FT-ICR Absorption CASI	DHB	Na	0.913	1668.86	3859.40
**Felodipine**	FT-ICR CASI	DHB	H	0.999	182.08	647.39
**Felodipine**	FT-ICR Magnitude	DHA	K	0.9623	313.45	611.13
**Felodipine**	FT-ICR Magnitude	DHA	Na	0.9565	391.28	676.31
**Felodipine**	FT-ICR Magnitude	DHA	H	0.9753	1279.90	2131.94
**Felodipine**	FT-ICR Magnitude	DHB	Na	0.9864	139.76	396.51
**Felodipine**	FT-ICR Magnitude	DHB	K	0.9773	155.59	404.24
**Felodipine**	FT-ICR Magnitude	DHB	H	0.994	300.22	966.11
**Felodipine**	FT-ICR Magnitude	FleX	Na	0.9943	57.32	283.75
**Felodipine**	FT-ICR Magnitude	FleX	K	0.9943	91.03	363.58
**Felodipine**	FT-ICR Magnitude	FleX	H	0.979	791.06	1729.93
**Felodipine**	MALDI-2	DHA	H	0.9838	716.98	1365.28
**Felodipine**	MALDI-2	DHA	Na	0.9813	925.23	1709.94
**Felodipine**	MALDI-2	DHA	K	0.9877	1689.87	3182.21
**Felodipine**	MALDI-2	DHB	H	0.9926	113.71	292.29
**Felodipine**	MALDI-2	DHB	Na	0.9954	188.66	408.65
**Felodipine**	MALDI-2	DHB	K	0.9924	320.12	674.41
**Felodipine**	MALDI-2	FleX	Na	0.9852	193.60	482.51
**Felodipine**	MALDI-2	FleX	K	0.9766	420.89	919.00
**Felodipine**	MALDI-2	FleX	H	0.9707	732.70	1568.19
**Felodipine**	MALDI-2 w/TIMS	DHA	H	0.9989	778.46	1364.08
**Felodipine**	MALDI-2 w/TIMS	DHA	K	0.999	1537.88	2573.21
**Felodipine**	MALDI-2 w/TIMS	DHA	Na	0.999	1139.91	3963.87
**Felodipine**	MALDI-2 w/TIMS	DHB	H	0.996	211.61	542.76
**Felodipine**	MALDI-2 w/TIMS	DHB	Na	0.997	354.70	759.87
**Felodipine**	MALDI-2 w/TIMS	DHB	K	0.9922	516.18	1192.20
**Felodipine**	MALDI-2 w/TIMS	FleX	Na	0.9963	225.01	551.40
**Felodipine**	MALDI-2 w/TIMS	FleX	K	0.9945	324.02	787.19
**Felodipine**	MALDI-2 w/TIMS	FleX	H	0.9936	670.50	1460.46

Target	Platform	Matrix	Cation	R^2	LoB (μg/g)	LoD (μg/g)
**MMAE**	AP-MALDI 6500	DHA	H	0.9812	13.52	29.08
**MMAE**	AP-MALDI 6500	DHB	H	0.9967	15.58	32.10
**MMAE**	AP-MALDI 6500	FleX	H	0.9812	13.55	28.38
**MMAE**	AP-MALDI 6500+	FleX	H	0.9956	0.32	3.69
**MMAE**	DESI Orbitrap	N/A	Na	0.9711	5.08	998.28
**MMAE**	DESI Orbitrap	N/A	H	0.9918	0.00	2629.05
**MMAE**	FT-ICR Absorption	DHB	K	0.9098	19.49	80.16
**MMAE**	FT-ICR Absorption	DHB	H	0.9007	121.82	215.98
**MMAE**	FT-ICR Absorption	DHB	Na	0.9038	182.59	330.26
**MMAE**	FT-ICR Absorption CASI	DHB	K	0.9968	19.54	59.66
**MMAE**	FT-ICR Absorption CASI	DHB	H	0.9926	56.33	118.86
**MMAE**	FT-ICR Absorption CASI	DHB	Na	0.9971	132.20	244.53
**MMAE**	FT-ICR CASI	DHB	K	0.9993	9.06	57.64
**MMAE**	FT-ICR CASI	DHB	H	0.9999	53.02	122.26
**MMAE**	FT-ICR CASI	DHB	Na	0.9988	125.62	195.12
**MMAE**	FT-ICR Magnitude	DHA	Na	0.9539	18.11	138.68
**MMAE**	FT-ICR Magnitude	DHA	H	0.9685	48.14	179.68
**MMAE**	FT-ICR Magnitude	DHA	K	0.9791	189.13	457.66
**MMAE**	FT-ICR Magnitude	DHB	Na	0.9984	62.15	142.92
**MMAE**	FT-ICR Magnitude	DHB	K	0.9994	84.67	154.29
**MMAE**	FT-ICR Magnitude	DHB	H	0.9962	151.50	328.34
**MMAE**	FT-ICR Magnitude	FleX	H	0.9939	7.04	51.15
**MMAE**	FT-ICR Magnitude	FleX	K	0.9993	64.38	128.06
**MMAE**	FT-ICR Magnitude	FleX	Na	0.9974	45.16	133.85
**MMAE**	MALDI-2	DHA	H	0.9956	62.97	99.56
**MMAE**	MALDI-2	DHA	K	0.9684	70.82	117.72
**MMAE**	MALDI-2	DHA	Na	0.9992	156.93	259.85
**MMAE**	MALDI-2	DHB	Na	0.9934	39.87	94.25
**MMAE**	MALDI-2	DHB	K	0.9994	87.95	190.36
**MMAE**	MALDI-2	DHB	H	0.9961	374.12	798.00
**MMAE**	MALDI-2	FleX	Na	0.9923	15.04	65.40
**MMAE**	MALDI-2	FleX	K	0.9943	37.16	84.00
**MMAE**	MALDI-2	FleX	H	0.9865	821.14	198.79
**MMAE**	MALDI-2 w/TIMS	DHA	H	0.9969	43.49	105.91
**MMAE**	MALDI-2 w/TIMS	DHA	K	0.9906	60.57	144.65
**MMAE**	MALDI-2 w/TIMS	DHA	Na	0.9966	137.23	282.54
**MMAE**	MALDI-2 w/TIMS	DHB	Na	0.9650	29.21	79.89
**MMAE**	MALDI-2 w/TIMS	DHB	K	0.9658	182.54	396.01
**MMAE**	MALDI-2 w/TIMS	DHB	H	0.9687	505.58	1119.96
**MMAE**	MALDI-2 w/TIMS	FleX	Na	0.9540	33.99	121.11
**MMAE**	MALDI-2 w/TIMS	FleX	K	0.9906	78.25	174.34
**MMAE**	MALDI-2 w/TIMS	FleX	H	0.9983	83.45	192.72
**VZ-185**	AP-MALDI 6500	DHA	H	0.9398	6.49	13.76
**VZ-185**	AP-MALDI 6500	DHB	H	0.8159	70.80	143.17
**VZ-185**	AP-MALDI 6500	FleX	H	0.9536	19.45	41.83
**VZ-185**	AP-MALDI 6500+	FleX	H	0.9941	3.64	7.69

**Target**	**Platform**	**Matrix**	**Cation**	**R^2**	**LoB (µg/g)**	**LoD (µg/g)**
**VZ-185**	DESI Orbitrap	N/A	Na	0.9264	2852.29	7126.02
**VZ-185**	DESI Orbitrap	N/A	H	0.9839	2204.88	10009.58
**VZ-185**	FT-ICR Absorption	DHB	H	0.9858	264.25	715.13
**VZ-185**	FT-ICR Absorption	DHB	Na	0.9711	4275.97	8376.63
**VZ-185**	FT-ICR Absorption	DHB	K	0.9716	6736.51	13373.87
**VZ-185**	FT-ICR Absorption CASI	DHB	H	0.9864	318.20	798.05
**VZ-185**	FT-ICR Absorption CASI	DHB	Na	0.9127	1668.86	3859.40
**VZ-185**	FT-ICR Absorption CASI	DHB	K	0.9438	7209.72	14598.83
**VZ-185**	FT-ICR CASI	DHB	H	0.9997	202.99	475.77
**VZ-185**	FT-ICR CASI	DHB	Na	0.9891	1878.14	4177.51
**VZ-185**	FT-ICR CASI	DHB	K	0.9733	1330.07	3191.49
**VZ-185**	FT-ICR Magnitude	DHA	H	0.9417	505.43	1370.84
**VZ-185**	FT-ICR Magnitude	DHB	H	0.9124	972.86	2125.36
**VZ-185**	FT-ICR Magnitude	FleX	H	0.9190	1787.43	3656.65
**VZ-185**	MALDI-2	DHA	H	0.9905	1519.94	2975.61
**VZ-185**	MALDI-2	DHB	H	0.9243	3019.26	6488.45
**VZ-185**	MALDI-2	FleX	H	0.9637	1879.81	3636.96
**VZ-185**	MALDI-2 w/TIMS	DHA	H	0.9655	816.73	1534.08
**VZ-185**	MALDI-2 w/TIMS	DHB	H	0.9791	1649.22	3565.95
**VZ-185**	MALDI-2 w/TIMS	FleX	H	0.9711	4439.45	8619.81

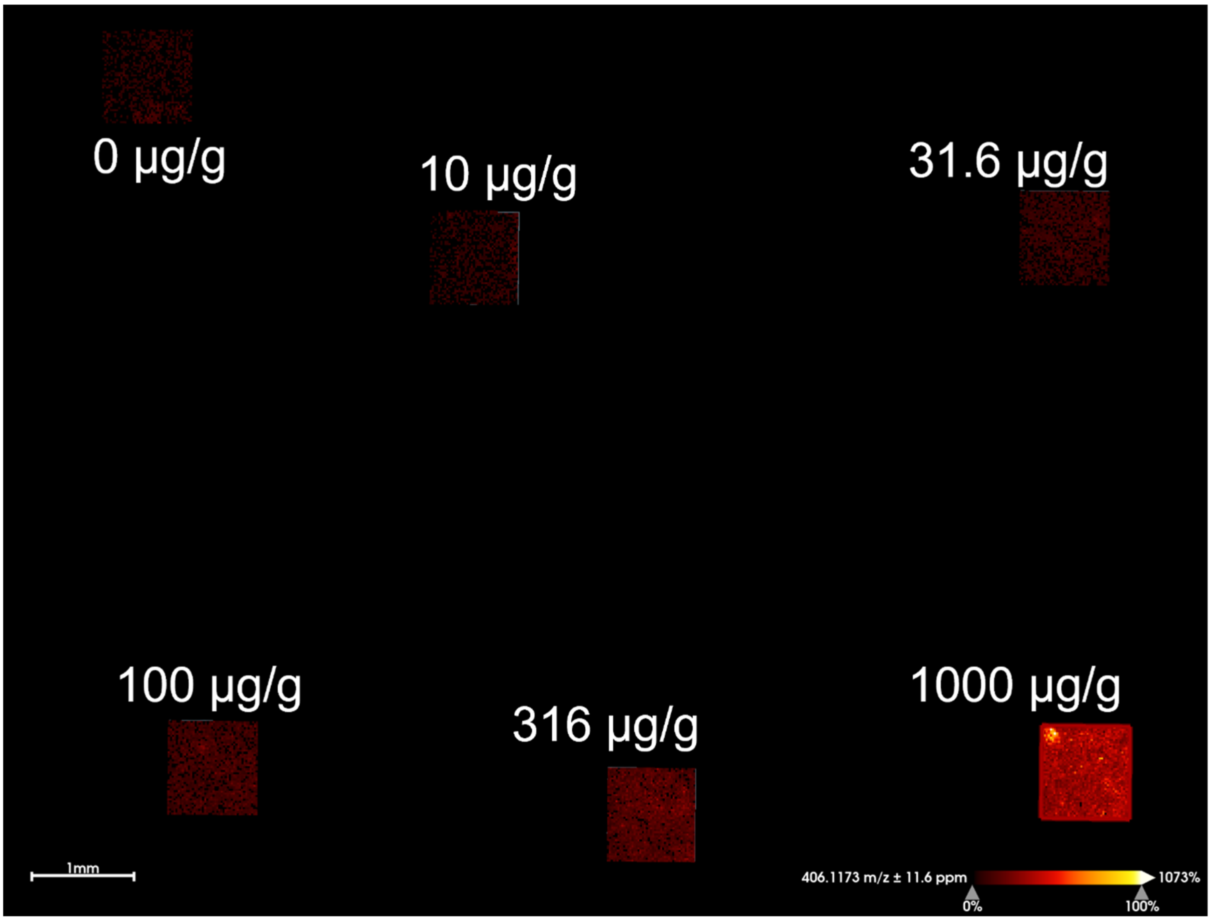

Supplementary Figure S1. Image of 6 concentrations of felodipine on TimsTOF FleX. Comparative image of all 6 concentrations of the small drug felodipine collected on the TimsTOF Flex, with both the MALDI-2 and TIMS options enabled. Images are relatively flat within each section, though there are occasional hotspots, especially visible in the upper left corner of 1000 µg/g.

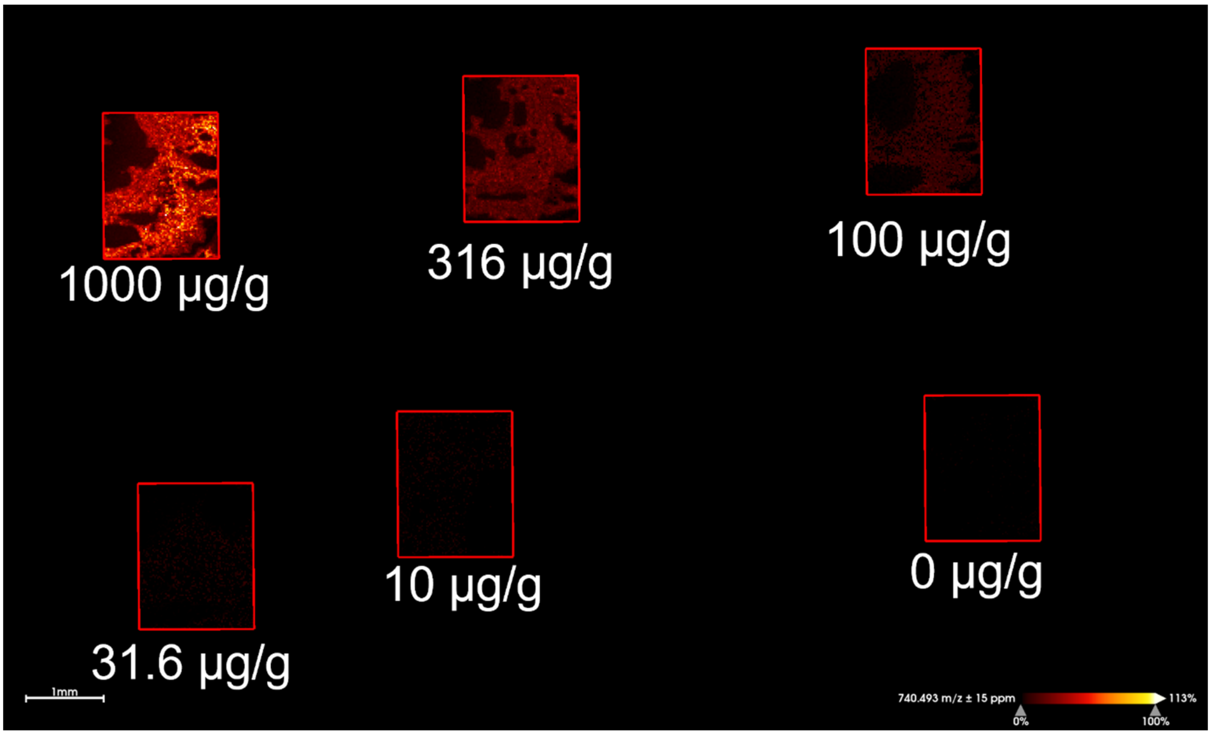

Supplementary Figure S2. Image of 6 concentrations of MMAE. Comparative image of all 6 concentrations of the antibody-drug conjugate monomethylauristatin E on the TimsTOF FleX, with only the MALDI-2 option enabled. Images are relatively homogeneous, though the larger image size shows the cracking problem that can occur with tissue mimetic models.

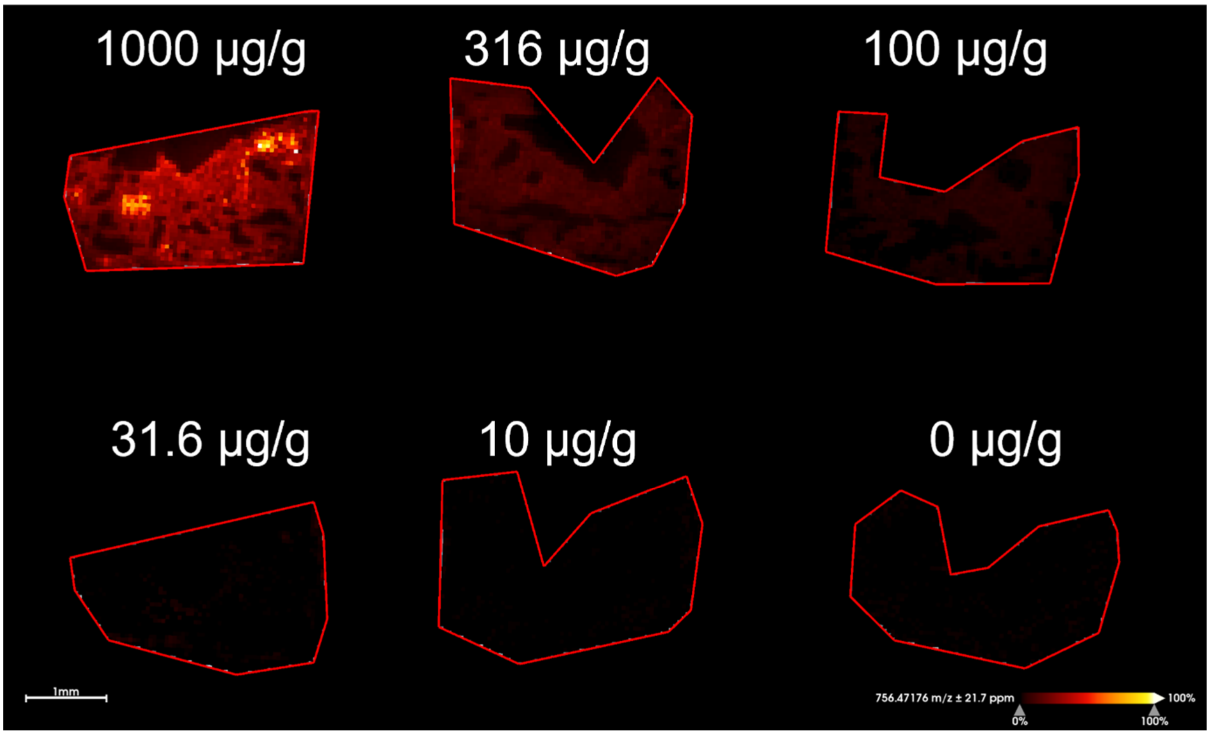

Supplementary Figure S3. Image of 6 concentrations of VZ-185 on SolariX. Comparative image of all 6 concentrations of the antibody-drug conjugate monomethylauristatin E on the SolariX 2xR. Images are relatively homogeneous, though the larger image size shows the cracking problem that can occur with tissue mimetic models and some hotspots are visible in the highest concentrations.

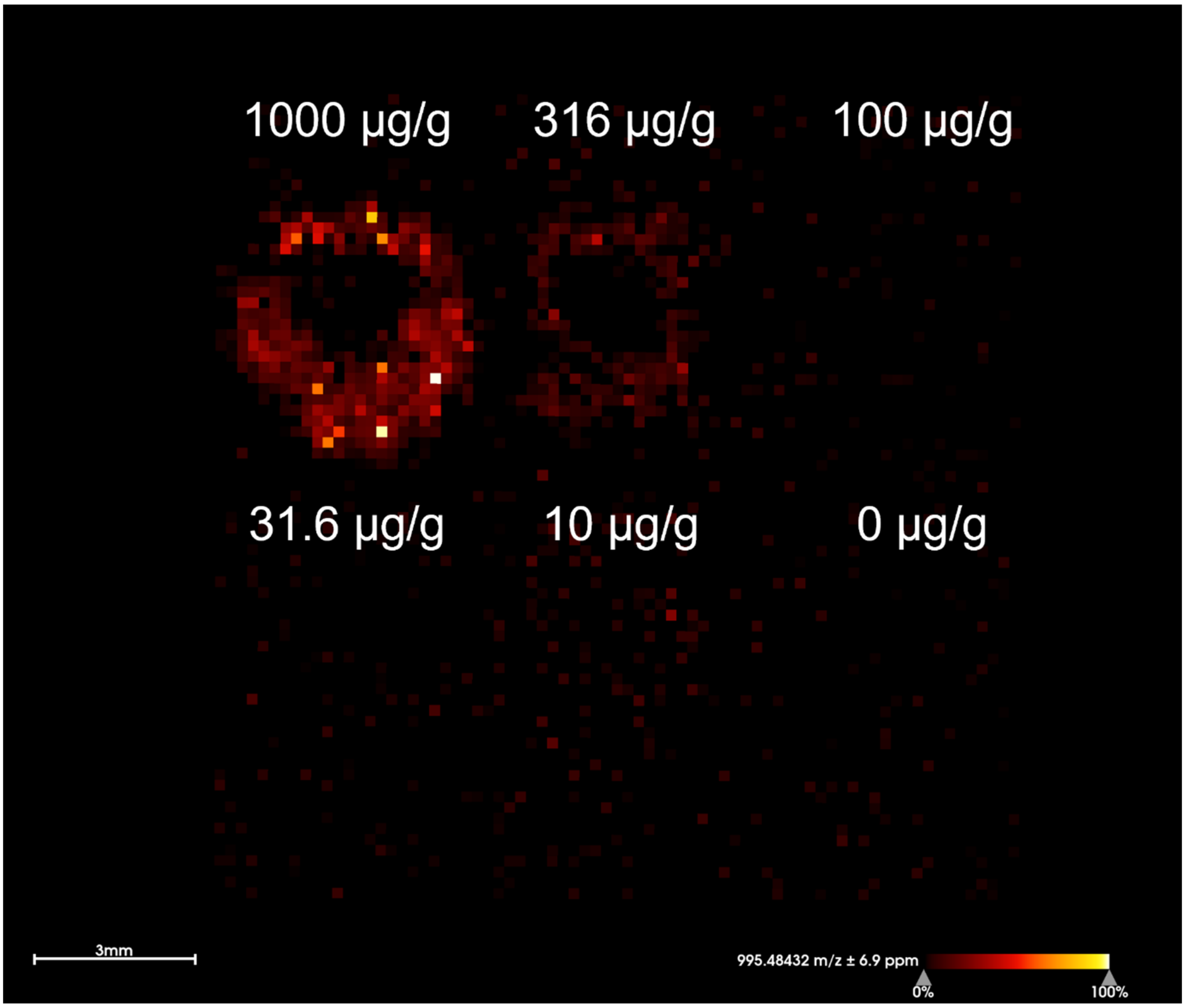

Supplementary Figure S4. Image of 6 concentrations of VZ-185 on DESI platform. Comparative image of all 6 concentrations of the protein degrader VZ-185 on the Q Exactive Plus system with a Prosolia DESI source. The tissue mimetic appears to have stuck to the inside of the gelatin mold more extensively, and the apparent homogeneity of the samples are less than available on the MALDI platforms.

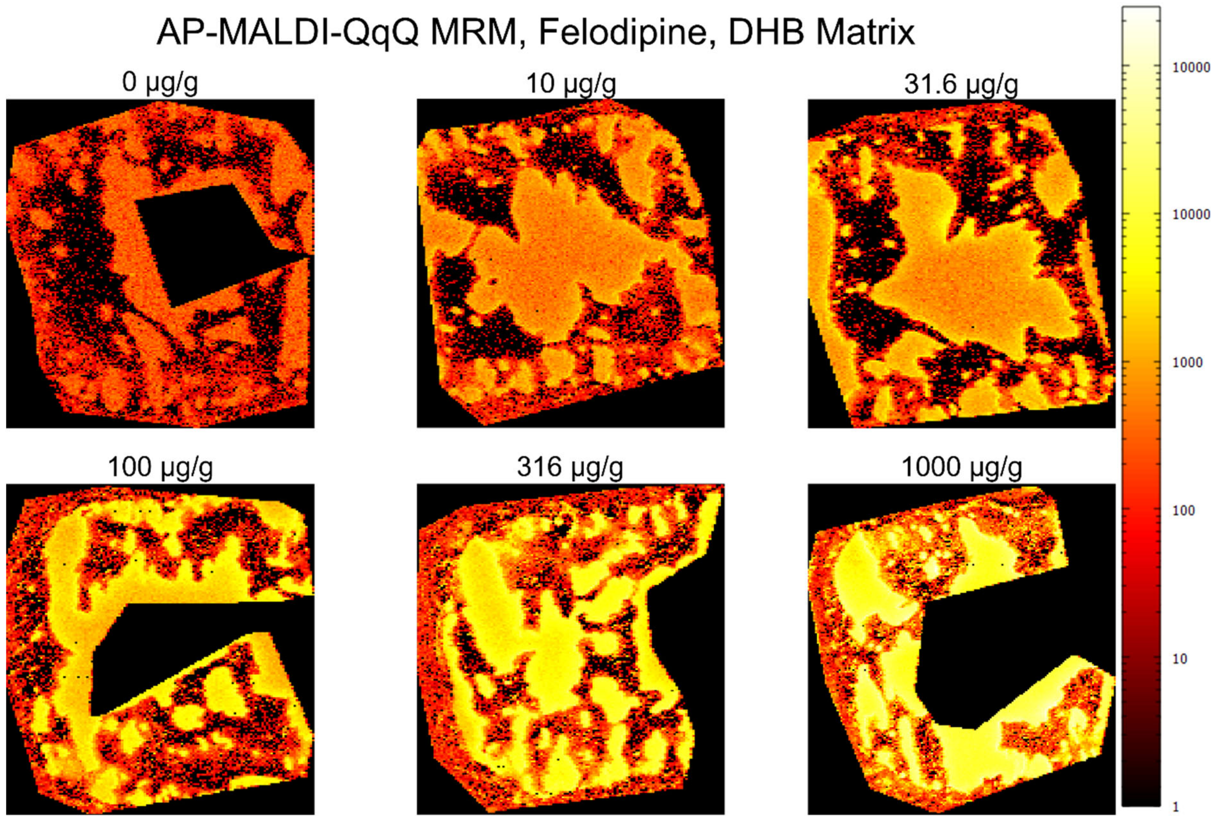

Supplementary Figure S5. Image of 6 concentrations of Felodipine on AP-MALDI-QQQ platform. Comparative image of all 6 concentrations of the small drug felodipine on the AB Sciex 6500 with a MassTech AP-MALDI UHR source. Images are relatively homogeneous, though the cracking problem that can occur with tissue mimetic models is readily apparent.

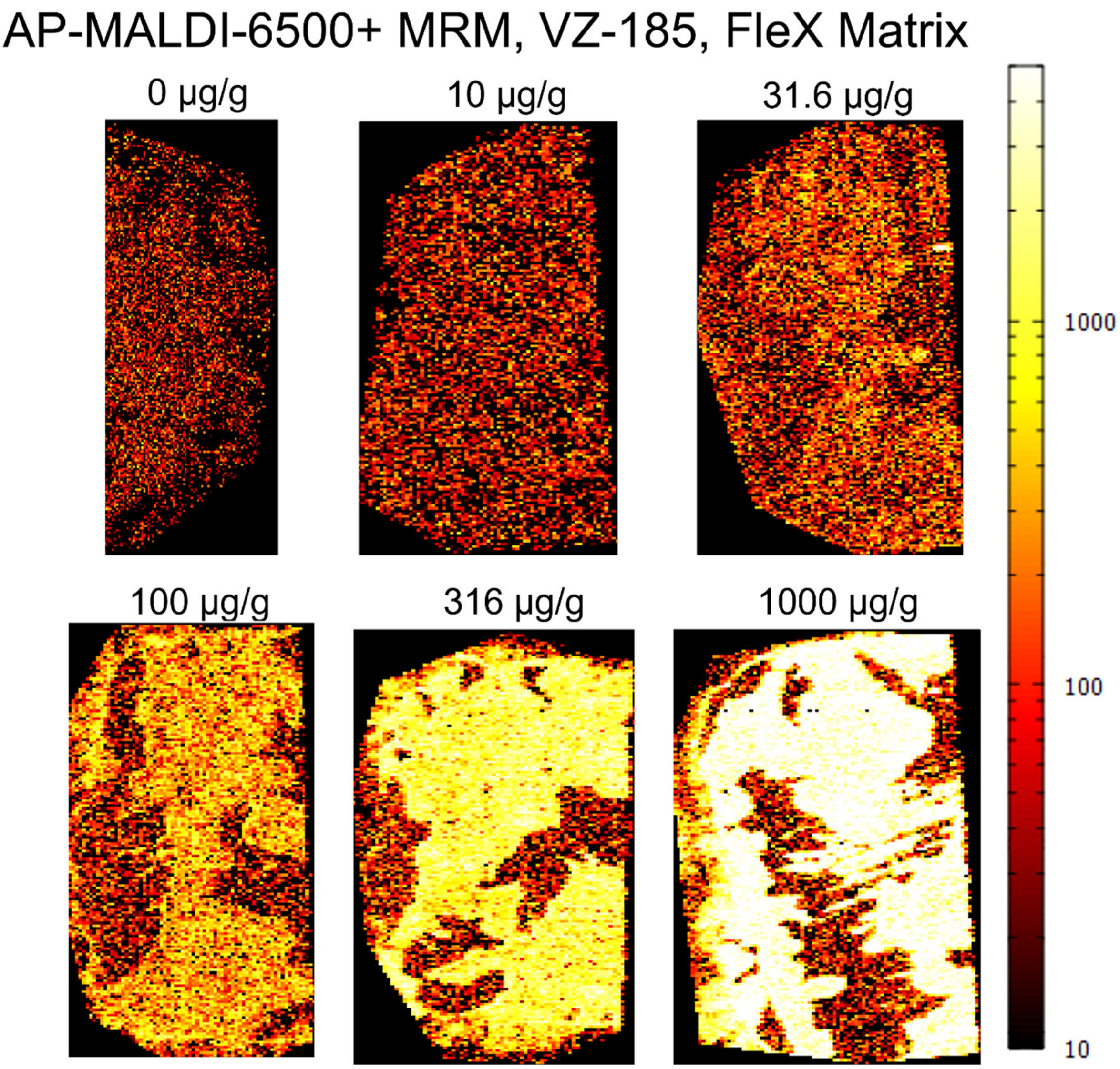

Supplementary Figure S6. Image of 6 concentrations of VZ-185 on AP-MALDI-QQQ platform Comparative image of all 6 concentrations of the protein degrader VZ-185 on the AB Sciex 6500+ with a MassTech AP-MALDI UHR source. Images are relatively homogeneous, though the cracking problem that can occur with tissue mimetic models is readily apparent. Inhomogeneity seems more apparent at 31.6 µg/g, and effectively non-existent by 1000 µg/g.

Supplementary Table S2. Average and Standard Deviation of LoB and LoD of Protonated Felodipine. Calculated average and standard deviation of 6 concentrations of protonated felodipine across 3 different times on a TimsTOF FleX with the MALDI-2 option enabled. Each slide was sectioned, had matrix applied, and was run on instrument separately. RSD of detected analyte concentration is 20.6% for the Limit of Detection, and 29.65% for the Limit of Blank.

Slide Number	Acquisition Date	Concentration (µg/g)	Mean (µg/g)	Standard Deviation		
**5**	2-May	0.00	6.18	8.06		
**5**	2-May	10.00	14.79	14.54		
**5**	2-May	31.60	16.96	13.82		
**5**	2-May	100.00	31.58	22.66		
**5**	2-May	316.00	47.84	30.34	Limit of Blank	234.37
**5**	2-May	1000.00	64.93	38.71	Limit of Detection	657.25
**15**	2-May	0.00	16.76	15.32		
**15**	2-May	10.00	22.32	17.37		
**15**	2-May	31.60	28.45	21.23		
**15**	2-May	100.00	36.56	23.23		
**15**	2-May	316.00	63.67	30.36	Limit of Blank	275.54
**15**	2-May	1000.00	120.64	41.88	Limit of Detection	587.82
**10**	5-Apr	0.00	11.67	14.68		
**10**	5-Apr	10.00	36.67	32.07		
**10**	5-Apr	31.60	53.76	44.52		
**10**	5-Apr	100.00	44.77	33.40		
**10**	5-Apr	316.00	101.84	67.66	Limit of Blank	114.04
**10**	5-Apr	1000.00	258.49	150.23	Limit of Detection	363.22
**1**	23-Mar	0.00	8.39	5.59		
**1**	23-Mar	10.00	10.76	7.17		
**1**	23-Mar	31.60	14.25	9.50		
**1**	23-Mar	100.00	17.50	11.66		
**1**	23-Mar	316.00	24.27	16.18	Limit of Blank	252.79
**1**	23-Mar	1000.00	50.41	33.61	Limit of Detection	577.07
**3**	23-Mar	0.00	15.58	23.19		
**3**	23-Mar	10.00	22.84	27.99		
**3**	23-Mar	31.60	25.80	27.71		
**3**	23-Mar	100.00	35.09	34.45		
**3**	23-Mar	316.00	72.54	48.61	Limit of Blank	284.08
**3**	23-Mar	1000.00	172.95	81.93	Limit of Detection	626.98
					Average Limit of Blank	232.17
					Average Limit of Detection	562.47
					Std Dev Limit of Blank	68.85
					Std Dev Limit of Detection	115.87
					Relative Std Dev Limit of Blank	29.65
					Relative Std Dev Limit of Detection	20.60

Supplementary Table S3. Average and Standard Deviation of LoB and LoD of Sodiated Felodipine. Calculated average and standard deviation of 6 concentrations of sodiated felodipine across 3 different times on a TimsTOF FleX with the MALDI-2 option enabled. Each slide was sectioned, had matrix applied, and was run on instrument separately. RSD of detected analyte concentration is 31.70% for the Limit of Detection, and 33.91% for the Limit of Blank.

Slide Number	Acquisition Date	Concentration (µg/g)	Mean (µg/g)	Standard Deviation		
**5**	2-May	0.00	4.83	12.09		
**5**	2-May	10.00	6.06	12.59		
**5**	2-May	31.60	13.22	19.75		
**5**	2-May	100.00	25.74	30.11		
**5**	2-May	316.00	53.14	44.52	Limit of Blank	126.66
**5**	2-May	1000.00	195.06	87.87	Limit of Detection	258.57
**15**	2-May	0.00	33.83	31.79		
**15**	2-May	10.00	38.30	42.15		
**15**	2-May	31.60	44.73	35.99		
**15**	2-May	100.00	70.13	41.50		
**15**	2-May	316.00	108.68	49.17	Limit of Blank	189.14
**15**	2-May	1000.00	372.10	120.45	Limit of Detection	439.96
**10**	5-Apr	0.00	22.67	13.24		
**10**	5-Apr	10.00	39.84	16.78		
**10**	5-Apr	31.60	41.23	18.97		
**10**	5-Apr	100.00	53.74	19.40		
**10**	5-Apr	316.00	71.65	19.38	Limit of Blank	155.61
**10**	5-Apr	1000.00	188.66	58.03	Limit of Detection	352.82
**1**	23-Mar	0.00	35.49	9.81		
**1**	23-Mar	10.00	41.26	10.46		
**1**	23-Mar	31.60	47.14	12.74		
**1**	23-Mar	100.00	50.03	12.27		
**1**	23-Mar	316.00	59.60	12.18	Limit of Blank	299.77
**1**	23-Mar	1000.00	97.26	12.36	Limit of Detection	619.46
**3**	23-Mar	0.00	45.90	16.25		
**3**	23-Mar	10.00	60.31	17.91		
**3**	23-Mar	31.60	71.08	19.83		
**3**	23-Mar	100.00	84.78	19.48		
**3**	23-Mar	316.00	102.80	19.04	Limit of Blank	234.42
**3**	23-Mar	1000.00	174.01	26.17	Limit of Detection	492.86
					Average Limit of Blank	201.12
					Average Limit of Detection	432.73
					Std Dev Limit of Blank	68.19
					Std Dev Limit of Detection	137.16
					Relative Std Dev Limit of Blank	33.91
					Relative Std Dev Limit of Detection	31.70

Supplementary Table S4. Average and Standard Deviation of LoB and LoD of Potassiated Felodipine. Calculated average and standard deviation of 6 concentrations of Potassiated felodipine across 3 different times on a TimsTOF FleX with the MALDI-2 option enabled. Each slide was sectioned, had matrix applied, and was run on instrument separately. RSD of detected analyte concentration is 26.35% for the Limit of Detection, and 28.23% for the Limit of Blank.

Slide Number	Acquisition Date	Concentration (µg/g)	Mean (µg/g)	Standard Deviation		
**5**	2-May	0.00	3.37	9.38		
**5**	2-May	10.00	5.06	11.32		
**5**	2-May	31.60	8.57	14.96		
**5**	2-May	100.00	15.91	21.53		
**5**	2-May	316.00	20.64	25.74	Limit of Blank	159.70
**5**	2-May	1000.00	124.42	68.31	Limit of Detection	352.36
**15**	2-May	0.00	33.51	16.76		
**15**	2-May	10.00	30.25	15.13		
**15**	2-May	31.60	30.53	15.26		
**15**	2-May	100.00	44.46	22.23		
**15**	2-May	316.00	54.05	27.02	Limit of Blank	263.86
**15**	2-May	1000.00	164.33	82.17	Limit of Detection	502.07
**10**	5-Apr	0.00	6.94	3.49		
**10**	5-Apr	10.00	8.92	3.90		
**10**	5-Apr	31.60	9.62	4.22		
**10**	5-Apr	100.00	13.28	4.73		
**10**	5-Apr	316.00	17.89	5.18	Limit of Blank	162.56
**10**	5-Apr	1000.00	49.49	15.68	Limit of Detection	344.06
**1**	23-Mar	0.00	18.54	5.25		
**1**	23-Mar	10.00	20.56	5.48		
**1**	23-Mar	31.60	23.51	6.05		
**1**	23-Mar	100.00	26.41	5.64		
**1**	23-Mar	316.00	38.23	6.54	Limit of Blank	233.77
**1**	23-Mar	1000.00	60.27	8.55	Limit of Detection	477.61
**3**	23-Mar	0.00	17.37	8.70		
**3**	23-Mar	10.00	24.66	9.58		
**3**	23-Mar	31.60	29.76	9.39		
**3**	23-Mar	100.00	34.13	10.99		
**3**	23-Mar	316.00	41.50	11.88	Limit of Blank	304.90
**3**	23-Mar	1000.00	69.76	19.06	Limit of Detection	640.52
					Average Limit of Blank	224.96
					Average Limit of Detection	463.32
					Std Dev Limit of Blank	63.51
					Std Dev Limit of Detection	122.11
					Relative Std Dev Limit of Blank	28.23
					Relative Std Dev Limit of Detection	26.35
